# Socioeconomic and lifestyle factors associated with depressive tendencies in general Japanese men and women: NIPPON DATA2010

**DOI:** 10.1186/s12199-019-0788-6

**Published:** 2019-05-28

**Authors:** Harumitsu Suzuki, Aya Kadota, Nagako Okuda, Takehito Hayakawa, Nobuo Nishi, Yasuyuki Nakamura, Hisatomi Arima, Naoko Miyagawa, Atsushi Satoh, Naomi Miyamatsu, Masahiko Yanagita, Hiroshi Yatsuya, Zentaro Yamagata, Takayoshi Ohkubo, Tomonori Okamura, Hirotsugu Ueshima, Akira Okayama, Katsuyuki Miura, for the NIPPON DATA2010 Research Group

**Affiliations:** 1 Center for Epidemiologic Research in Asia, Shiga University of Medical Science, Tsukinowa-cho, Seta, Otsu, Shiga 520-2192 Japan; 2 Department of Hygiene, Wakayama Medical University, Wakayama, Japan; 3 Department of Public Health, Shiga University of Medical Science, Tsukinowa-cho, Seta, Otsu, Shiga 520-2192 Japan; 4 Department of Health and Nutrition, University of Human Arts and Sciences, Saitama, Japan; 5 The Kinugasa Research Organization, Ritsumeikan University, Kyoto, Japan; 6 International Center for Nutrition and Information, National Institute of Health and Nutrition, National Institutes of Biomedical Innovation, Health and Nutrition, Tokyo, Japan; 7 Department of Food Sciences and Human Nutrition, Ryukoku University, Otsu, Japan; 8 Faculty of Medicine, School of Medicine, Fukuoka University, Fukuoka, Japan; 9 Department of Clinical Nursing, Shiga University of Medical Science, Otsu, Japan; 10 Department of Health and Sports Science, Doshisha University, Kyotanabe, Japan; 11 Department of Public Health, Fujita Health University School of Medicine, Toyoake, Japan; 12 Department of Health Sciences, Yamanashi University, Kofu, Japan; 13 Department of Hygiene and Public Health, Teikyo University, Tokyo, Japan; 14 Department of Preventive Medicine and Public Health, Keio University, Tokyo, Japan; 15 Research Institute of Strategy for Prevention, Tokyo, Japan

**Keywords:** Depression, Gender, Socioeconomic factors, Lifestyle, Social psychiatry, Japanese

## Abstract

**Background:**

The gender-specific characteristics of individuals at an increased risk of developing depression currently remain unclear despite a higher prevalence of depression in women than in men. This study clarified socioeconomic and lifestyle factors associated with an increased risk of subclinical depression in general Japanese men and women.

**Methods:**

Study participants were residents not receiving psychiatric treatments in 300 sites throughout Japan in 2010 (1152 men, 1529 women). Multivariable-adjusted odds ratios (OR) and 95% confidence intervals (95%CIs) for socioeconomic factors and lifestyle factors were calculated using a logistic regression analysis.

**Results:**

Risk of depressive tendencies was significantly higher in men who were single and living alone (OR, 3.27; 95% CI, 1.56–6.88) than those married. The risk was significantly lower in women who were not working and aged ≥ 60 years (OR, 0.39; 95% CI, 0.22–0.68) and higher in men who were not working and aged < 60 years (OR, 3.57; 95%CI, 1.31–9.72) compared with those who were working. Current smoking was also associated with a significantly increased risk of depressive tendencies in women (OR, 2.96; 95% CI, 1.68–5.22) but not in men.

**Conclusions:**

Socioeconomic and lifestyle factors were associated with an increased risk of depressive tendencies in general Japanese. Related factors were different by sex.

**Electronic supplementary material:**

The online version of this article (10.1186/s12199-019-0788-6) contains supplementary material, which is available to authorized users.

## Introduction

Recent studies showed the prevalence of mental disorders in Japan remained stable and treatment rate has increased for those who have moderate disorders in the last decade [1, 2]. Although, the number of patients with mood disorders increased from 433,000 in 1996 to 1,116,000 in 2014 according to a survey conducted by the Japanese Ministry of Health, Labour, and Welfare [3, 4], treatment rate was still lower compared to the USA and more than 60% of people with moderate/mild disorders did not have any treatment [2]. Depression has become a social issue that needs comprehensive measures. A study using meta-analysis showed that non-specific and specific intervention studies in individuals without depression at baseline reduced the risk of depression after follow-up periods [5].

The early detection and prevention of disease progression are one of the potential strategies for depression. To figure out any characteristics of individuals associated with subclinical depression among the general population, it may be useful for public or industrial healthcare professionals to find and support those at high risk. Previous studies reported the characteristics of individuals at an increased risk of developing depression, including marital status, living arrangement, and income [2, 6–9]. However, the gender-specific characteristics of individuals at an increased risk of developing depression currently remain unclear despite an approximately twofold higher prevalence of depression in women than in men [10, 11]. Therefore, we hypothesized that there were differences in socioeconomic and lifestyle determinants to be associated with subclinical depression by sex in general Japanese. These characteristics need to be clarified separately by sex, age, and working status in order to formulate effective measures in a super-aging society due to elderly people at high risk [9, 12].

National Integrated Project for Prospective Observation of Non-communicable Disease and its Trends in the Aged (NIPPON DATA) 2010 is a cohort study based on the National Health and Nutrition Survey Japan in 2010. Participants were residents from 300 survey districts selected throughout Japan. In the present study, we used baseline survey data from NIPPON DATA2010 to investigate relationships between socioeconomic and lifestyle factors and depressive tendencies as assessed by Kessler 6 (K6) among this best available representative Japanese population.

## Methods

### Study population

The study population comprised the participants of a prospective cohort study of cardiovascular diseases named the National Integrated Project for Prospective Observation of Non-communicable Disease and its Trends in the Aged (NIPPON DATA) 2010 [13]. Participants were men and women aged 20 years and older who resided in 300 randomly selected areas throughout Japan and participated in the National Health and Nutrition Survey (NHNS) in 2010, which was conducted by the Ministry of Health, Labour, and Welfare [14]. Among 3873 participants aged 20 years or older who had a blood test in NHNS, 2898 participants (74.6%) agreed to participate in the cohort study, NIPPON DATA2010. They also participated in the Comprehensive Survey of Living Conditions (CSLC) in 2010, which was also conducted by the Ministry of Health, Labour, and Welfare; data from CSLC 2010 were merged to NIPPON DATA2010 dataset. The methods of NHNS, CSLC, and NIPPON DATA2010 have been described in detail elsewhere [13–15]. Trained interviewers obtained informed consent before enrollment. The Institutional Review Board of the Shiga University of Medical Science approved this study (No. 22–29, 2010).

A total of 164 participants were excluded because it was not possible to merge data from NHNS 2010 and CSLC 2010 with NIPPON DATA2010 baseline data (*n* = 91) or because of missing data (*n* = 73). Additional 53 patients were excluded because they were receiving treatments for psychiatric diseases. The remaining 2681 participants (1152 men, 1529 women) were included in the present study (Additional file 1: Figure S1).

### Socioeconomic status

Information on socioeconomic factors was collected using the self-administered questionnaires of NIPPON DATA2010 (family status), NHNS 2010 (working status), and CSLC 2010 (monthly household expenditure of 2010 May, the month before CSLC 2010, home ownership, and health insurance).

The following socioeconomic factors were considered in the present study. Family status was grouped into three strata: “married,” “single living with family,” “single living alone.” “Single” included those who never married, were widowed, and divorced. Working status was grouped according to three categories: “working,” “not working aged < 60 years,” and “not working aged ≥ 60 years.” Those not working included students and homemakers. We categorized not working participants into two groups at the age of 60 years because most companies in Japan set a compulsory retirement age of 60 years for indefinite-term employees. Equivalent monthly household expenditure was calculated as household expenditure divided by the square root of the number of household members. Participants were categorized into quartiles using equivalent household expenditure. Home ownership was used to adjust the equivalent monthly household expenditure, because, in the CSLC questionnaire, rent in non-home owners was taken into account as part of expenditure, but mortgage payment in home owners was not. Participants who answered that they “did not know the household expenditure” were included as “no answer” in the analysis. Regarding health insurance, participants were categorized into three groups: national health insurance, employee’s health insurance, and other/no answer. National health insurance is managed by municipal governments and insures self-employed individuals, his/her families, and not working individuals. Participants insured by insurance systems for the elderly aged 75 years or older were included in this strata. Employee’s health insurance insures workers at companies and public offices, including their families. Other/no answer included participants who had received public livelihood assistance or had not answered the question.

### Lifestyle and other information

Public health nurses collected information on smoking (current, ex-, or non-smoker), drinking habit, and a previous history of stroke, myocardial infarction, or angina pectoris using a standardized questionnaire in NHNS. Regarding drinking habits, participants were asked about the frequency of alcohol consumption per week and the typical amount consumed per day expressed in “gou” (Japanese unit of alcohol, with 1 gou being equivalent to 23 g of ethanol). Average alcohol consumption (gou/day) was calculated as the number of alcohol drinking days per week multiplied by the amount of alcohol per day, then divided by 7 days. Drinking habits were categorized into four strata: non-drinker, ex-drinker, current drinker < 2 gou/day, current drinker ≥ 2 gou/day. Participants who answered that they had a history of stroke, myocardial infarction, or angina pectoris were identified as “with a history of cardiovascular disease (CVD).”

### K6 scale

The Japanese version of the K6 scale was obtained in the self-administered questionnaire for NIPPON DATA2010. K6 consists of 6 questions, which ask “during the past 30 days, how often did you feel the following: nervous, hopeless, restless, or fidgety, so depressed that nothing could cheer you up, that everything was an effort, and worthless”, scored as “0: None of the time”, “1: A little of the time”, “2: Some of the time”, “3: Most of the time” and “4: All of the time”, with a total score of 0–24 points [16, 17]. A higher number indicated distress. In the present study, depressive tendencies were identified by a K6 score ≥ 9 based on a previous validation study suggesting a score for identifying individuals at a high risk of depressive symptoms [18, 19].

### Statistical analysis

All analyses were performed in total and by sex. The characteristics of participants with and without depressive tendencies were compared using *t* test and chi-squared test for continuous and categorical variables, respectively. The relationships between socioeconomic and lifestyle factors and depressive tendencies were examined using a sex-age-adjusted logistic regression analysis. A multivariable logistic regression analysis was performed to assess the independent relationships between socioeconomic and lifestyle factors and depressive tendencies. Sex, age, family status, working status, equivalent monthly household expenditure and home ownership, health insurance, lifestyle factors (drinking and smoking habits), and a history of CVD were used as explanatory variables.

The results of logistic regression analyses were reported as odds ratios (ORs) with corresponding 95% confidence intervals (95%CIs). *P* <  0.05 was considered to be significant. All analyses were performed using SAS software version 9.4 for Windows (SAS Institute Inc., Cary, NC, USA).

## Results

### Participant characteristics

Table 1 shows the characteristics of participants with or without depressive tendencies. A total of 193 (7.2%) participants had depressive tendencies (K6 ≥ 9) (67 men [5.8%], 126 women [8.2%]). Participants with depressive tendencies were around 4 years younger than those without in both men and women. Men with depressive tendencies included fewer married men. Furthermore, no differences were observed in the family status between women with and without depressive tendencies. Working status was statistically different by depressive tendencies in both men and women. Fewer women participants with depressive tendencies were insured by the national health insurance. Regarding lifestyle factors, more current smokers were included with depressive tendencies among women. No differences were observed in drinking habits and a previous history of CVD in men and women.

**Table 1:** Characteristics of study participants stratified by the depressive status with the Kessler 6 scale. Japanese men and women aged 20 years and older. NIPPON DATA2010 baseline survey (*n* = 2681)

	Men			Women		
	K6 < 9 (*n* = 1085)	K6 ≥ 9 (*n* = 67)	*P*	K6 < 9 (*n* = 1403)	K6 ≥ 9 (*n* = 126)	*P*
	*n* (%)	*n* (%)		*n* (%)	*n* (%)	
Age, years*	60.3 (15.4)	56.7 (16.8)	0.07	58.3 (16.0)	54.4 (14.8)	0.007
Family status						
Married	895 (82.5)	44 (65.7)	0.001	1032 (73.6)	90 (71.4)	0.803
Single living with family	102 (9.4)	10 (14.9)		214 (15.2)	22 (17.5)	
Single living alone	88 (8.1)	13 (19.4)		157 (11.2)	14 (11.1)	
Working status						
Working	689 (63.5)	42 (62.7)	< 0.001	575 (41.0)	71 (56.3)	< 0.001
Not working aged < 60 years	23 (2.1)	7 (10.4)		235 (16.7)	24 (19.1)	
Not working aged ≥ 60 years	373 (34.4)	18 (26.9)		593 (42.3)	31 (24.6)	
Equivalent household expenditure per month (10^4^ JPY)^†^	12.7 (8.9–17.5)	11.5 (8.8–15.6)	0.808	13.3 (9.2–17.5)	14.1 (8.8–17.9)	0.250
Health insurance						
National	602 (55.5)	34 (50.7)	0.553	724 (51.6)	59 (46.8)	0.012
Employee’s	466 (42.9)	31 (46.3)		660 (47.0)	61 (48.4)	
Other/no answer	17 (1.6)	2 (3.0)		19 (1.4)	6 (4.8)	
Drinking status						
Non-drinker	261 (24.1)	22 (32.8)	0.325	882 (62.9)	77 (61.1)	0.151
Ex-drinker	34 (3.1)	2 (3.0)		17 (1.2)	0 (0)	
< 2 gou^‡^/day	652 (60.1)	33 (49.3)		489 (34.8)	45 (35.7)	
≥ 2 gou/day	138 (12.7)	10 (14.9)		15 (1.1)	4 (3.2)	
Smoking status						
Non-smoker	379 (34.9)	25 (37.3)	0.104	1245 (88.8)	97 (77.0)	< 0.001
Ex-smoker	420 (38.7)	18 (26.9)		82 (5.8)	9 (7.1)	
Current smoker	286 (26.4)	24 (35.8)		76 (5.4)	20 (15.9)	
History of cardiovascular disease ^§^						
No	970 (89.4)	55 (82.1)	0.064	1336 (95.2)	118 (93.7)	0.433
Yes	115 (10.6)	12 (17.9)		67 (4.8)	8 (6.3)	

*K6*, Kessler 6; *JPY*, Japanese yen; *IQR*, interquartile range

*Age was expressed as mean (SD)

^†^Equivalent household expenditure per month (10^4^ JPY) was calculated as monthly household expenditure divided by the square root of the number of family members and expressed as median (IQR)

^‡^Unit of alcohol beverage equivalent to 23 g of ethanol

^§^Stroke, myocardial infarction, or angina pectoris

### Relationships between socioeconomic/lifestyle factors and depressive tendencies

Sex-age-adjusted ORs for socioeconomic/lifestyle factors associated with depressive tendencies are shown in Table 2. Women had a higher OR compared with men. Participants who were single and living alone had a significantly higher OR for depressive tendencies than those who were married. Participants who were not working aged ≥ 60 years had a lower OR than those who were working. Regarding health insurance, the OR of depressive tendencies was higher categorized as “other/no answer” than among those insured by the National Health Insurance. Regarding lifestyle factors, a significantly elevated OR was observed for current smokers. Participants with a history of CVD had a higher OR for depressive tendencies than those without a history of CVD. Age-adjusted ORs for socioeconomic/lifestyle factors associated with depressive tendencies by sex are shown in Additional file 2: Table S2. Men had a higher OR for “single living alone,” “not working aged < 60 years,” and “history of CVD.” Women had higher OR for “other/no answer” of category in the health insurance and for current smoker and had lower OR for “not working aged ≥ 60 years.”

**Table 2:** Socioeconomic/lifestyle factors and sex-age-adjusted odds ratios of depressive tendencies (Kessler 6 ≥ 9) in Japanese men and women aged 20 years and older. NIPPON DATA2010 baseline survey (*n* = 2681)

	*n*/total *n* (%)	Odds ratio (95% CI)
Sex		
Men	67/1152 (5.8)	Reference
Women	126/1529 (8.2)	1.41*^†^ (1.04–1.92)
Family status		
Married	134/2061 (6.5)	Reference
Single living with family	32/348 (9.2)	1.23 (0.81–1.86)
Single living alone	27/272 (9.9)	1.74* (1.12–2.71)
Working status		
Working	113/1377 (8.2)	Reference
Not working aged < 60 years	31/289 (10.7)	1.11 (0.72–1.74)
Not working aged ≥ 60 years	49/1015 (4.8)	0.59* (0.38–0.90)
Equivalent household expenditure per month^‡^		
Q1	46/601 (7.7)	1.31 (0.85–2.03)
Q2	41/671 (6.1)	Reference
Q3	49/654 (7.5)	1.25 (0.81–1.92)
Q4	45/625 (7.2)	1.23 (0.79–1.90)
No answer	12/130 (9.2)	1.58 (0.80–3.09)
Health insurance		
National	93/1419 (6.6)	Reference
Employee’s	92/1218 (7.6)	0.83 (0.58–1.18)
Other/no answer	8/44 (18.2)	2.81* (1.26–6.28)
Drinking status		
Non-drinker	99/1242 (8.0)	Reference
Ex-drinker	2/53 (3.8)	0.53 (0.13–2.21)
< 2 gou^§^/day	78/1219 (6.4)	0.82 (0.59–1.13)
≥ 2 gou/day	14/167 (8.4)	1.22 (0.65–2.29)
Smoking status		
Non-smoker	122/1746 (7.0)	Reference
Ex-smoker	27/529 (5.1)	0.99 (0.61–1.59)
Current smoker	44/406 (10.8)	1.97* (1.30–2.99)
History of cardiovascular disease ^‖^		
No	173/2479 (7.0)	Reference
Yes	20/202 (9.9)	2.07* (1.24–3.47)

*CI*, confidence interval

^*^P value < 0.05 was considered to be significant

^†^Odds ratio was adjusted for age only

^‡^Calculated as monthly household expenditure divided by square root of the number of family members

^§^Unit of alcohol beverage equivalent to 23 g of ethanol

^‖^Stroke, myocardial infarction, or angina pectoris

We then performed a multivariable logistic regression analysis to identify independent relationships. Because there were sex interactions in some factors (Additional file 2: Table S1), analyses were done in total participants and in each sex group (Table 3). The risk of depressive tendencies was significantly higher in “single living alone” than “married” in total participants and in men. It was lower for “not working participants aged ≥ 60 years” in total participants and in women than for those who were working. The risk was significantly increased for current smokers in total participants and in women and for those who had a history of CVD in total participants and in men. In addition, the risk of depressive tendencies was significantly higher for women than for men. When we included participants who were receiving psychiatric treatments, the results obtained from the analysis were similar (data not shown).

**Table 3:** Socioeconomic/lifestyle factors and multivariable adjusted odds ratios^¶^ of depressive tendencies (Kessler 6 ≥ 9) in Japanese men and women aged 20 years and older. NIPPON DATA2010 baseline survey (*n* = 2681)

	Total	Men	Women
	OR (95% CI)	OR (95% CI)	OR (95% CI)
Sex			
Men	Reference		
Women	1.74* (1.17–2.60)		
Family status			
Married	Reference	Reference	Reference
Single living with family	1.19 (0.77–1.83)	1.41 (0.61–3.25)	1.02 (0.60–1.71)
Single living alone	1.65* (1.03–2.65)	3.27* (1.56–6.88)	1.08 (0.57–2.07)
Working status			
Working	Reference	Reference	Reference
Not working aged < 60 years	1.15 (0.73–1.80)	3.57* (1.31–9.72)	0.85 (0.51–1.42)
Not working aged ≥ 60 years	0.52* (0.33–0.82)	0.90 (0.44–1.88)	0.39* (0.22–0.68)
Equivalent household expenditure per month^‡^			
Q1	1.25 (0.80–1.95)	0.97 (0.47–1.98)	1.50 (0.84–2.68)
Q2	Reference	Reference	Reference
Q3	1.30 (0.84–2.00)	1.06 (0.52–2.16)	1.51 (0.86–2.66)
Q4	1.27 (0.81–1.99)	0.63 (0.29–1.39)	1.77* (1.00–3.13)
No answer	1.52 (0.77–3.00)	2.09 (0.74–5.90)	1.22 (0.47–3.17)
Health insurance			
National	Reference	Reference	Reference
Employee’s	0.79 (0.55–1.13)	1.12 (0.60–2.09)	0.68 (0.43–1.07)
Other/no answer	2.54* (1.10–5.83)	1.16 (0.23–5.83)	3.68* (1.34–10.1)
Drinking status			
Non-drinker	Reference	Reference	Reference
Ex-drinker	0.59 (0.14–2.52)	0.95 (0.20–4.41)	NA
< 2 gou^§^/day	0.82 (0.59–1.15)	0.71 (0.40–1.28)	0.91^†^ (0.61–1.35)
≥ 2 gou/day	1.11 (0.58–2.11)	1.09 (0.49–2.47)	
Smoking status			
Non-smoker	Reference	Reference	Reference
Ex-smoker	1.00 (0.62–1.63)	0.62 (0.32–1.20)	1.42 (0.67–2.99)
Current smoker	1.89* (1.24–2.90)	1.07 (0.58–1.97)	2.96* (1.68–5.22)
History of cardiovascular disease ^‖^			
No	Reference	Reference	Reference
Yes	2.15* (1.28–3.64)	2.10* (1.01–4.35)	1.82 (0.82–4.08)

*OR*, odds ratio; *CI*, confidence interval; *NA*, not available

*P value < 0.05 was considered to be significant

^†^Due to small number, two categories (> 2 gou/day and ≥ 2 gou/day) were combined

^‡^Calculated as monthly household expenditure divided by square root of the number of family members

^§^Unit of alcohol beverage equivalent to 23 g of ethanol

^‖^Stroke, myocardial infarction, or angina pectoris

^¶^All variables and house ownership are included simultaneously in the same model

## Discussion

We examined relationships between socioeconomic and lifestyle factors and depressive tendencies in a general Japanese population in the baseline survey for NIPPON DATA2010, which included young and elderly, and working and not working, participants who resided in 300 randomly selected areas throughout Japan. While men who were single and living alone had a significantly higher risk of depressive tendencies than married men, this relationship was not found for women. Men not working aged < 60 years had a higher risk of depressive tendencies than working men, while women not working aged ≥ 60 years had a decreased risk. To the best of our knowledge, this is the first study to have examined depressive tendencies and associated factors in the best available representative population sample of Japanese men and women.

The higher prevalence of depression in women has been explained by sex hormone change and social roles in Japan [20, 21] and in Western countries [11]. In Western countries, an approximately twofold higher prevalence of depression in women than in men was reported since 1977 [22]. A recent research in Japan showed twofold higher prevalence in 2004–2006 [23], although older researches in Japan could not find the differences. These findings suggest that the social environment has been changing in Japan.

Labor participation rates have been increasing among women, who had typically stayed home to raise children in the period of economic growth in the twentieth century [12, 24]. Accordingly, men and women delayed marriage or stayed unmarried, which resulted in an increase in the percentage of unmarried people and one-person households [25]. Many working women have been classified as part-time workers with a low salary [26, 27]; the gender gap has remained greater in Japan than in other developed countries [28]. Even among married women, social support for childcare and the associated costs are limited for working women [12] and short sleep duration was related to depressive symptoms in women but not in men [29]. Working and household work in women might make sleep duration shorter.

The number of patients with depressive disorders per 100,000 population increased in Japan between 1996 and 2014 from 1.6 to 4.2 for men and from 2.7 to 7.0 for women [3, 4]. According to a Statistics Bureau, Ministry of Internal Affairs and Communications, report from 1996 to 2014, employment rate of the working age population among 15–64-year olds has increased from 56.8 to 64.5% for women, while it has reached a plateau at 80% for men [30]. Changes in the family status of men and increased labor participation among women may have contributed to the increases in the number of patients with depression. Even for the mortality, men who were unmarried or living alone among workers had higher risk [31]. Supportive measures for men at risk of disconnected living and for women of working age with social and in-home support for housework, which may include raising children, would be important.

Total participants who were categorized as “other/no answer” for health insurance had an increased risk of developing depression. “Other” included those receiving public livelihood assistance, and “no answer” may have included those who did not have valid health insurance because of non-payment of the premium. Thus, participants who had lost income for health reasons may have been included in this category.

Smoking was associated with an increased risk of developing depression in women, but not in men. A relationship between cigarette use and depressive symptoms has been consistently reported [32], while gender differences in this relationship remain inconclusive [33, 34]. Smoking rates in women are lower in Japan than in Western countries. Social pressure against women’s smoking may be strong in Japan, and female smokers are more likely to be nicotine-dependent than male smokers, thereby contributing to the different results obtained between men and women.

We used a K6 questionnaire with 6 brief questions that were easy to answer and that has been used as a tool for the mass screening of depression [35]. The Japanese version was validated for use in seven communities in Japan, demonstrating an excellent area under the curve of 0.94 (95%CI 0.88–0.99), which was similar to other questionnaires with more question items for assessing mood and anxiety disorders in the Diagnostic and Statistical Manual of Mental Disorders (DSM-IV). A cut-off score of ≥ 9 in K6 for identifying subjects at an increased risk of developing depression was suggested according to a validation report, for which sensitivity and specificity were estimated as 77.8 and 86.4 in Japanese [18, 19], which were similar to other questionnaires with more question items [36]. We identified factors associated with depressive tendencies in a survey performed in up to 300 survey districts using the brief K6 questionnaire.

The strength of the present study was its representativeness of a Japanese study population from 300 randomly selected areas throughout Japan for NHNS 2010, including men and women, the young and elderly, and working and not working participants. Information on health insurance was included because the dataset was merged with CSLC 2010. There were several limitations in the present study. A cross-sectional analysis cannot estimate causal relationships between factors and depressive tendencies. Therefore, based on the present results, we cannot conclude these factors caused the development of depression. The possibility of a selection bias cannot be excluded because those who participated in all three surveys were included and the participation rate was not sufficiently high. There might have unmeasured confounding factors which we could not consider in our analyses. Trauma experience and low social supports were reported to be the risk factors of depression, and neurophysiological change in the brain could be associated with depression [11, 37]. Furthermore, we defined depressive tendencies using the K6 scale only, which may have resulted in misclassification.

## Conclusion

In conclusion, we herein identified potential factors associated with depressive tendencies in the best available representative Japanese population. Factors such as family status, working status, and smoking habit were related to depressive tendencies and sex differences existed in their associations. Our findings suggest the high-risk groups whom should be screened for depressive tendencies using such as K6 questionnaires.

## Additional files

Additional file 1: **Figure S1.** Study participants of NIPPON DATA2010 and selection flow. (PPTX 42 kb)

Additional file 2: **Table S1.** Age-adjusted sex interaction on the relationship between socioeconomic/lifestyle factors and depressive tendencies (Kessler 6 ≥ 9). **Table S2.** Socioeconomic/lifestyle factors and age-adjusted odds ratios of depressive tendencies (Kessler 6 ≥ 9). (DOCX 23 kb)

## Abbreviations

95%CIs: 95% confidence intervals
CSCL: Comprehensive Survey of Living Conditions
CVD: Cardiovascular disease
DSM-IV: Diagnostic and Statistical Manual of Mental Disorders
K6: Kessler 6
NHNS: National Health and Nutrition Survey
NIPPON DATA: National Integrated Project for Prospective Observation of Non-communicable Disease and its Trends in the Aged
OR: Odds ratios
