# Supplementary material for: Socioeconomic and lifestyle factors associated with depressive tendencies in general Japanese men and women: NIPPON DATA2010
Source: Environ Health Prev Med. 2019 May 28;24:37. doi: 10.1186/s12199-019-0788-6 (PMC6540356; doi:10.1186/s12199-019-0788-6)
Supplement: Supplementary file 1 — Figure S1. Study participants of NIPPON DATA2010 and selection flow. (PPTX 42 kb) [file 12199_2019_788_MOESM1_ESM.pptx]

## Slide 1
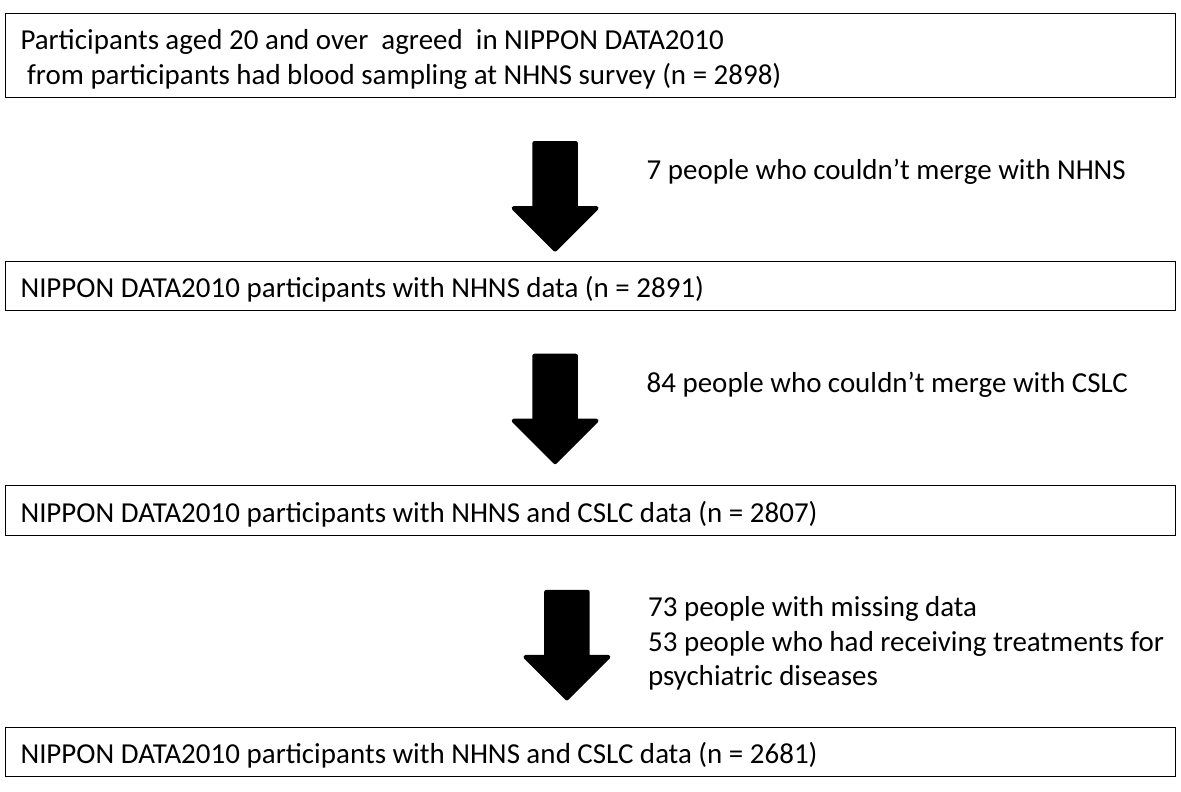

Participants aged 20 and over agreed in NIPPON DATA2010
 from participants had blood sampling at NHNS survey (n = 2898)
7 people who couldn’t merge with NHNS
NIPPON DATA2010 participants with NHNS data (n = 2891)
84 people who couldn’t merge with CSLC
NIPPON DATA2010 participants with NHNS and CSLC data (n = 2807)
73 people with missing data
53 people who had receiving treatments for
psychiatric diseases
NIPPON DATA2010 participants with NHNS and CSLC data (n = 2681)
