# Supplementary material for: Socioeconomic and lifestyle factors associated with depressive tendencies in general Japanese men and women: NIPPON DATA2010
Source: Environ Health Prev Med. 2019 May 28;24:37. doi: 10.1186/s12199-019-0788-6 (PMC6540356; doi:10.1186/s12199-019-0788-6)
Supplement: Supplementary file 2 — Table S1. Age-adjusted sex interaction on the relationship between socioeconomic/lifestyle factors and depressive tendencies (Kessler 6 ≥ 9). Table S2. Socioeconomic/lifestyle factors and age-adjusted odds ratios of depressive tendencies (Kessler 6 ≥ 9). (DOCX 23 kb) [file 12199_2019_788_MOESM2_ESM.docx]

Table S1. Age adjusted sex interaction on the relationship between socioeconomic/lifestyle factors and depressive tendencies (Kessler 6 ≥ 9). Japanese men and women aged 20 years and older. NIPPON DATA2010 baseline survey (n = 2681)

|  | β | *P* |  |
| --- | --- | --- | --- |
| Family status |  |  |  |
| Married | Reference | |  |
| Single living with family | -0.35 | 0.431 |  |
| Single living alone | -0.86 | 0.059 |  |
| Working status |  |  |  |
| Working | Reference | |  |
| Not working aged < 60 years | -1.79 | < 0.001 |  |
| Not working aged ≥ 60 years | -0.62 | 0.09 |  |
| Equivalent household expenditure per month ^†^ | |  | |
| Q1 | 0.48 | 0.294 |  |
| Q2 | Reference | |  |
| Q3 | 0.36 | 0.427 |  |
| Q4 | 0.89 | 0.066 |  |
| No answer | -0.58 | 0.404 |  |
| Health insurance |  |  |  |
| National | Reference | |  |
| Employee's | -0.06 | 0.854 |  |
| Other/no answer | 0.65 | 0.476 |  |
| Drinking status |  |  |  |
| Non-drinker | Reference | |  |
| Ex-drinker | -12.2 | 0.974 |  |
| < 2 gou^‡^/day | 0.44 | 0.211 |  |
| ≥ 2 gou/day | 1.13 | 0.109 |  |
| Smoking status |  |  |  |
| Non-smoker | Reference | |  |
| Ex-smoker | 0.61 | 0.217 |  |
| Current smoker | 0.90 | 0.027 |  |
| History of cardiovascular disease ^§^ |  |  |  |
| No | Reference | |  |
| Yes | -0.22 | 0.669 |  |

†, Calculated as monthly household expenditure divided by square root of the number of family members

‡, Unit of alcohol beverage equivalent to 23 g of ethanol

§, Stroke, myocardial infarction, or angina pectoris

Table S2. Socioeconomic/lifestyle factors and age-adjusted odds ratios of depressive tendencies (Kessler 6 ≥ 9). Japanese men and women aged 20 years and older. NIPPON DATA2010 baseline survey (n = 2681）

|  | Men | | |  | Women | | |
| --- | --- | --- | --- | --- | --- | --- | --- |
|  | n / total n (%) | aOR | (95% CI) |  | n / total n (%) | aOR | (95% CI) |
| Family status |  |  |  |  |  |  |  |
| Married | 44/939 (4.7) | Reference | |  | 90/1122 (8.0) | Reference | |
| Single living with family | 10/112 (8.9) | 1.68 | (0.78-3.62) |  | 22/236 (9.3) | 1.10 | (0.67-1.81) |
| Single living alone | 13/101 (12.9) | 2.93* | (1.52-5.66) |  | 14/171 (8.2) | 1.26 | (0.68-2.31) |
| Working status |  |  |  |  |  |  |  |
| Working | 42/731 (5.7) | Reference | |  | 71/646 (11.0) | Reference | |
| Not working aged < 60 years | 7/30 (23.3) | 4.63* | (1.85-11.6) |  | 31/624 (5.0) | 0.82 | (0.50-1.35) |
| Not working aged ≥ 60 years | 18/391 (4.6) | 0.94 | (0.47-1.86) |  | 24/259 (9.3) | 0.43* | (0.25-0.74) |
| Equivalent household expenditure per month ^‡^ | |  | |  |  |  | |
| Q1 | 16/268 (6.0) | 0.98 | (0.49-1.96) |  | 30/333 (9.6) | 1.59 | (0.90-2.80) |
| Q2 | 18/295 (6.1) | Reference | |  | 23/376 (6.1) | Reference | |
| Q3 | 16/272 (5.9) | 1.00 | (0.50-2.01) |  | 33/382 (8.6) | 1.44 | (0.83-2.51) |
| Q4 | 11/269 (4.1) | 0.68 | (0.32-1.48) |  | 34/356 (9.6) | 1.67 | (0.96-2.91) |
| No answer | 6/48 (12.5) | 2.23 | (0.84-5.97) |  | 6/82 (7.3) | 1.25 | (0.49-3.19) |
| Health insurance |  |  |  |  |  |  |  |
| National | 34/636 (5.3) | Reference | |  | 59/783 (7.5) | Reference | |
| Employee's | 31/497 (6.2) | 0.89 | (0.50-1.61) |  | 61/721 (8.5) | 0.79 | (0.51-1.24) |
| Other/no answer | 2/19 (10.5) | 1.85 | (0.41-8.42) |  | 6/25 (24.0) | 3.46* | (1.32-9.10) |
| Drinking status |  |  |  |  |  |  |  |
| Non-drinker | 22/283 (7.8) | Reference | |  | 77/959 (8.0) | Reference | |
| Ex-drinker | 2/36 (5.6) | 0.80 | (0.18-3.57) |  | 0/17 (0.0) | NA | NA |
| < 2 gou^§^/day | 33/685 (4.8) | 0.60 | (0.34-1.05) |  | 45/553 (8.1) | 0.98^†^ (0.66-1.44) | |
| ≥ 2 gou/day | 10/148 (6.8) | 0.84 | (0.38-1.82) |  |  |  |  |
| Smoking status |  |  |  |  |  |  |  |
| Non-smoker | 25/404 (6.2) | Reference | |  | 97/1342 (7.2) | Reference | |
| Ex-smoker | 18/438 (4.1) | 0.69 | (0.37-1.29) |  | 9/91 (9.9) | 1.26 | (0.61-2.61) |
| Current smoker | 24/310 (7.7) | 1.21 | (0.68-2.18) |  | 20/96 (20.8) | 2.98* | (1.72-5.17) |
| History of cardiovascular disease ^‖^ |  |  |  |  |  |  |  |
| No | 55/1025 (5.4) | Reference | |  | 118/1454 (8.1) | Reference | |
| Yes | 12/127 (9.4) | 2.32* | (1.16-4.62) |  | 8/75 (10.7) | 1.81 | (0.83-3.98) |

aOR, age-adjusted odd ratios; NA, not available

*, *P*-value < 0.05 was considered to be significant.

†, Due to small number, two categories ( > 2 gou/day and ≥ 2 gou/day) were combined.

‡, Calculated as monthly expenditure divided by the square root of the number of family members

§, Unit of alcohol beverage equivalent to 23 g of ethanol

‖, Stroke, myocardial infarction, or angina pectoris
